# Automated Detection of HONcode Website Conformity Compared to Manual Detection: An Evaluation

**DOI:** 10.2196/jmir.3831

**Published:** 2015-06-02

**Authors:** Célia Boyer, Ljiljana Dolamic

**Affiliations:** ^1^ Health On the Net Foundation Chêne-Bourg Switzerland

**Keywords:** HONcode, classification, artificial intelligence, natural language processing, quality standards

## Abstract

**Background:**

To earn HONcode certification, a website must conform to the 8 principles of the HONcode of Conduct In the current manual process of certification, a HONcode expert assesses the candidate website using precise guidelines for each principle. In the scope of the European project KHRESMOI, the Health on the Net (HON) Foundation has developed an automated system to assist in detecting a website’s HONcode conformity. Automated assistance in conducting HONcode reviews can expedite the current time-consuming tasks of HONcode certification and ongoing surveillance. Additionally, an automated tool used as a plugin to a general search engine might help to detect health websites that respect HONcode principles but have not yet been certified.

**Objective:**

The goal of this study was to determine whether the automated system is capable of performing as good as human experts for the task of identifying HONcode principles on health websites.

**Methods:**

Using manual evaluation by HONcode senior experts as a baseline, this study compared the capability of the automated HONcode detection system to that of the HONcode senior experts. A set of 27 health-related websites were manually assessed for compliance to each of the 8 HONcode principles by senior HONcode experts. The same set of websites were processed by the automated system for HONcode compliance detection based on supervised machine learning. The results obtained by these two methods were then compared.

**Results:**

For the privacy criterion, the automated system obtained the same results as the human expert for 17 of 27 sites (14 true positives and 3 true negatives) without noise (0 false positives). The remaining 10 false negative instances for the privacy criterion represented tolerable behavior because it is important that all automatically detected principle conformities are accurate (ie, specificity [100%] is preferred over sensitivity [58%] for the privacy criterion). In addition, the automated system had precision of at least 75%, with a recall of more than 50% for contact details (100% precision, 69% recall), authority (85% precision, 52% recall), and reference (75% precision, 56% recall). The results also revealed issues for some criteria such as date. Changing the “document” definition (ie, using the sentence instead of whole document as a unit of classification) within the automated system resolved some but not all of them.

**Conclusions:**

Study results indicate concordance between automated and expert manual compliance detection for authority, privacy, reference, and contact details. Results also indicate that using the same general parameters for automated detection of each criterion produces suboptimal results. Future work to configure optimal system parameters for each HONcode principle would improve results. The potential utility of integrating automated detection of HONcode conformity into future search engines is also discussed.

##  Introduction

The Internet has brought about immense change in the way individuals obtain and access health information [1]. It transformed health information distribution from occurring only in the doctor’s office during patient visits (top-down information flow) to a multilateral, asynchronous form of communication. Patients feel empowered to gather and share their own information and to make more informed decisions regarding their own health care [2,3].

A recent study showed that 35% of US adults had used the Internet at one time or another to gather health information about a medical condition that they or someone else had [4]. Of these Internet users, 46% had also sought the advice of a health professional. Conversely, 38% of persons accessing the Internet for health information stated that they managed the health condition at home. Given that more than 30% of US adults have made important health care decisions after accessing the Internet, the quality of Internet-based health information becomes crucial. Another recent study shows that, not less than 60% of Europeans go online when looking for health information [5]. Six out of 10 (60%) Europeans who have found health-related information online thought the information came from a trustworthy source although it remains unclear what they deemed as trustworthy [6].

However, taking into account the quantity of the health-related information available on the Internet in the form of health-related websites or scientific articles, users are often overwhelmed with the quantity of the information available. Recently, efforts have been taken to automatically label online health pages according to the information quality provided on them [7,8]. These research studies remain connected to a certain health domain and to quality criteria defined by study authors. Studies indicate that the quality of the health information found on the Internet is extremely variable [9,10]. Readers have exceeding difficulty in discerning trustworthy from nontrustworthy website content. One approach to this dilemma is to annotate websites that willingly comply to content quality with easily visible badges or icons. This is the approach taken by the Health on the Net (HON) Foundation in HONcode certification [11]. The HONcode is a code of conduct consisting of 8 procedural principles (ie, authority, complementarity, privacy, attribution, justification, contact details, financial disclosure, and advertising policy) that a health website must follow to gain certification [12]. The goal of this process is to create a pool of quality health information available to the general public [13,14]. The HONcode helps the Web user to judge if she/he can trust the information found on the Internet [15,16]. However, because obtaining HONcode certification requires a website manager to voluntarily submit a request for HON review, the scope of existing HONcode certification remains limited.

Search engines represent the source most frequently used. In one survey, 77% of online health advice seekers began their last session at a generalized search engine such as Google, Bing, or Yahoo [17]. A recent European study shows that between 82% and 87% of those who searched for health-related information online used search engines to do so [6]. These search engines typically list results according to popularity rather than quality or trustworthiness. Thus, the first few options they display may not be the best sources of health information. People become confused and anxious after accessing inappropriate health information [18]. Ideally, search engine developers would modify the search engine to promote the most reliable and validated sources of health information. Within the European project KHRESMOI (2010-2014, project No. 2575284), researchers have recently developed tools to automatically assess how well a given website complies with the HONcode principles. Complementing the authors’ and our colleagues’ work in developing the algorithm [19,20], this study presents an evaluation comparing automated detection of HONcode principle compliance with expert assessments for 30 health websites.

## Methods

### Overview

In this study, the authors compared the results of the automated system detection for HONcode principles for a selection of 30 health websites to the ones obtained during the standard manual HONcode process conducted by senior HONcode experts (eg, an expert with more than 10 years’ experience in HONcode certification). The senior HONcode expert has a medical background; he/she is responsible for training of new HONcode reviewers and deals with complex certification cases.

### HONcode Certification Process

Once a site has requested HONcode certification, the expert navigates the pages of the site to identify if the site respects each of the HONcode principles [12].

**Figure 1:**
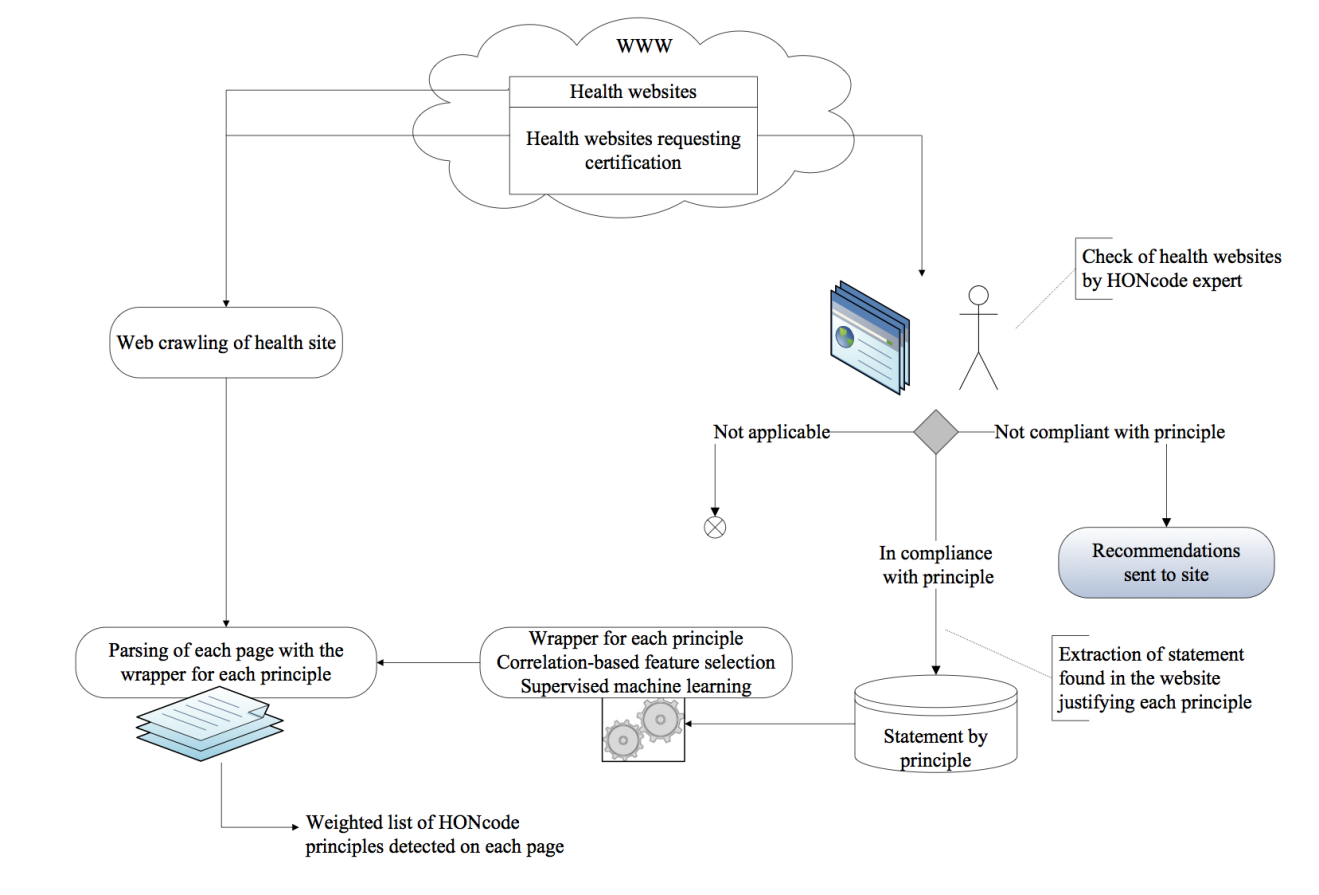
HONcode manual and automated detection processes.

When principle justification is found on a page (ie, the site conforms to the given principle), the extract and the Web address are added to the HONcode file and stored in a database. When a principle is not respected either totally or partially, recommendations are sent to the site editor. The manual HONcode certification is described in Figure 1.

### HONcode Interreviewer Agreement

With the goal of measuring the level of agreement between expert reviewers and estimating the likelihood of an expert giving a false assessment, we compared the assessments done by 3 senior reviewers for a total of 36 websites. Each criterion was rated by all 3 reviewers.

### Description of Automated System for HONcode Detection

Automated detection of HONcode principle compliance consisted of the following steps as illustrated in Figure 1:

1. For a given health-related website, a WebCrawler retrieved a maximal set of its accessible Web pages. This proceeded from the website home page and followed the internal links.
2. The system extracted “meaningful content” from the retrieved Web pages within a given website. This content consisted of textual information within the pages.
3. The content extracted from each Web page was then checked by the automated system for compliance with each HONcode principle. The automated system embodied the machine learning framework described in Williams and Calvo [21]. Separate classifiers were built for each of the HON criteria. The classifiers reviewed the Web page material independently because when a document indicated compliance with 1 HONcode principle, it did not exclude the possibility that the document complied with other HON principles (“any-of” classification) [22]. The process of automated HONcode detection was designed in this way to mimic the current manual certification process. However, the automated system systemically checks all the sites’ webpages retrieved unlike the manual system that stops once criterion compliance is detected. The extracts justifying principle compliance collected during HONcode certification formed the training set for the aforementioned classifiers. HONcode certification is multilingual; 34% of certified websites are in English, 28% in French, 10% in Spanish, and 7% in German. However, this study was limited to the English language only. The number of training documents varied from 872 for the criteria “justifiability” classifier to 2861 for the “contact details” classifier. The general classifier system enabled the user to select from different machine learning algorithms, such as naive Bayes, support vector machine (SVM), and others through various parameter settings [23]. The system also enabled choice of different feature types, such as bag-of-words, bag-of-stems, co-occurrence, etc. Additionally, the system implemented a user-configurable variety of feature selection algorithms (term weighting schemata). In this study, the authors specified use of the naive Bayes algorithm for each of the 8 HONcode principles. The algorithm as implemented checked the page content according to 9 different criteria because 1 of the 8 individual HONcode principles (“attribution”) was divided into 2 parts, “references” and “date,” for this study based on previously validated reasons [19].

To specify conversion of document word counts into vector values, the authors used 2 weighting schemes, namely tfc and tfx, in which *t*, *f*, *c*, and *x* represent document frequency, inverse document frequency, cosine normalization, and none, respectively [24]. The document frequency (t) represents the number of occurrences of the given term within the document being classified. The inverse document frequency (f) is calculated as f=log(N/D), where N is total number of documents within the collection and D represents the number of documents in the collection that contain the given term. Thus, a higher importance is given to a term found in a smaller number of documents within the collection, supposing that the more the documents the term is found in, the less important it is. The final variables indicate whether cosine normalization occurs (c) versus none (x). This parameter gives more importance to the term occurrences within shorter documents. Thus, the tfc conversion additionally normalizes the score by the document length (c).

### Automated System Detection Results Compared to the Manual Evaluation Results

The authors selected a convenience sample of 30 health care websites for the comparative evaluation (automated detection vs manual rating by a senior HONcode expert). However, only 27 of 30 websites could be processed by the automated system, so study results used the sample of 27 sites. The convenience sample was selected to broadly cover HONcode potential and actual sites as follows:

1. New potentially certifiable websites (n=9): the HONcode experts estimated that these websites did conform to HONcode, but they had not yet been certified.
2. Likely noncertifiable websites (n=9): the HONcode experts estimated that these websites would not conform to HONcode principles when fully analyzed.
3. Newly certified websites (n=4): these websites had been recently certified for the first time.
4. Previously certified HONcode sites (n=5): these websites were chosen because they were awaiting annual reassessment.

**Figure 2:**
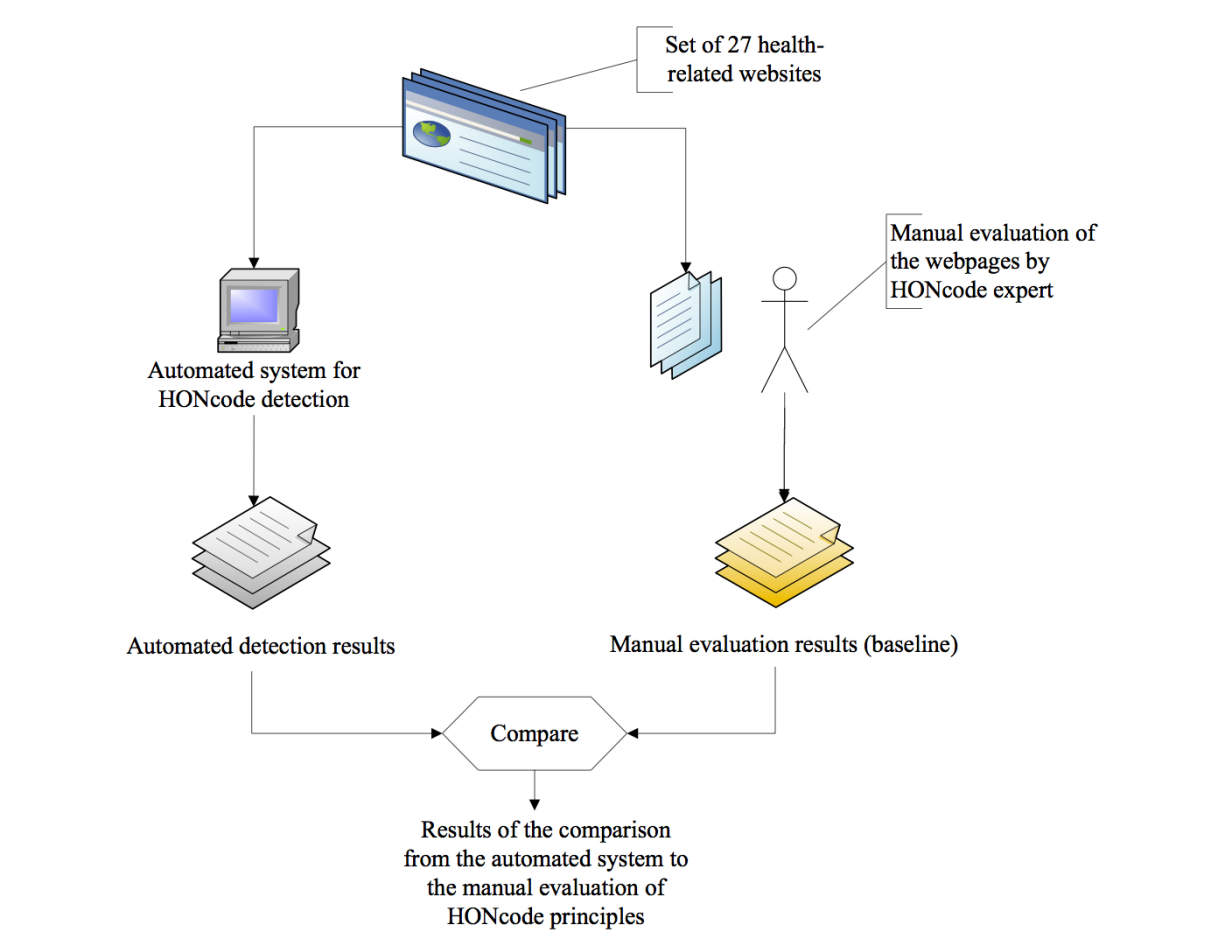
Comparison of the automated HONcode detection evaluation to manual evaluation.

For the purpose of the evaluation, the senior HONcode expert manually reviewed each of the 27 websites described. Simultaneously, the automated system for HONcode detection reviewed the 27 websites for each evaluation criterion [19]. The results obtained by the automated system were then compared to the baseline obtained by the expert. Figure 2 shows the evaluation methods.

**Figure 3:**
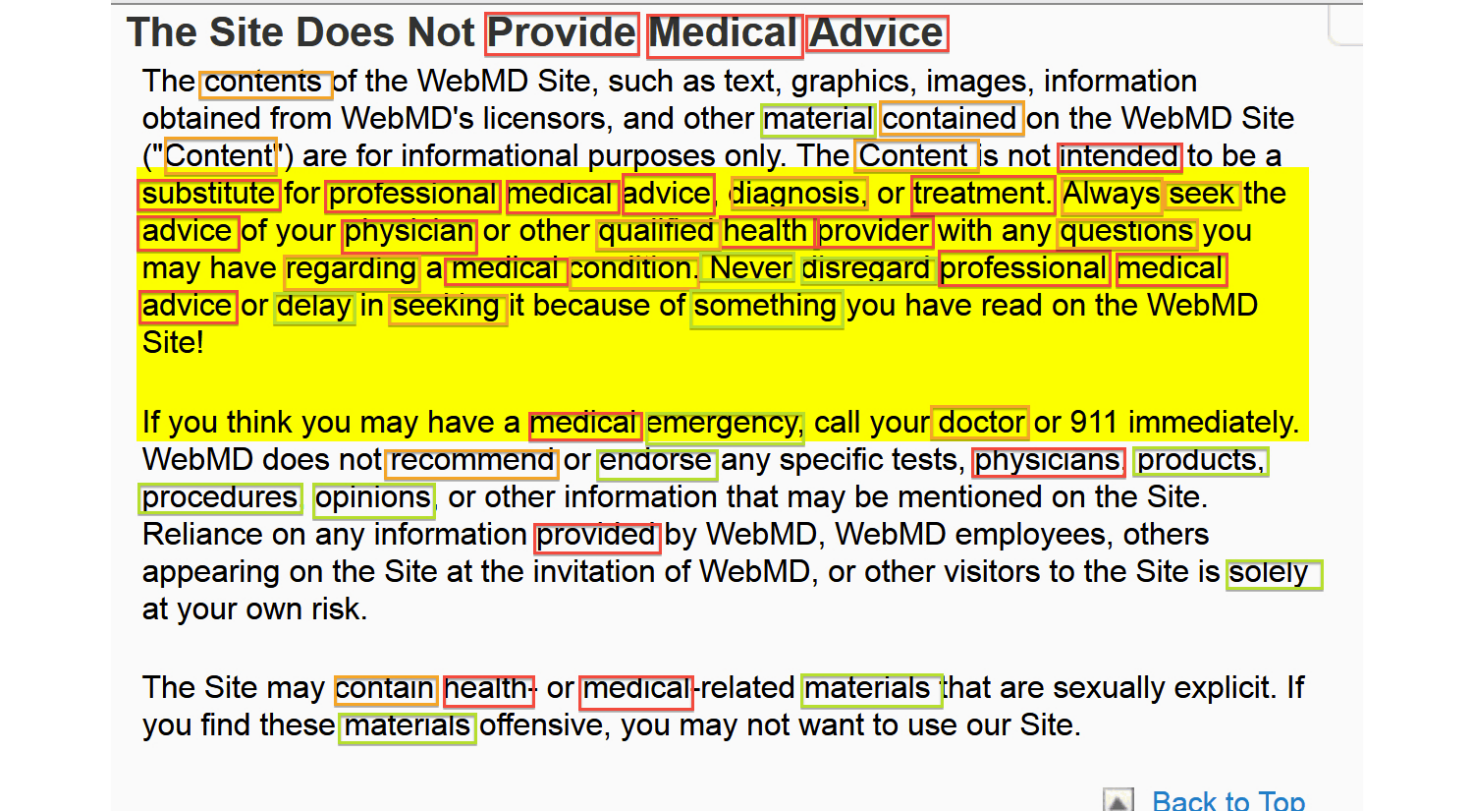
Assessment of “complementarity” criterion with terms detected by the expert (highlighted in yellow) and the automated system (colored boxes with red=most important and green=least important).

Figure 3 gives a sample page conforming to the “complementarity” criterion. On this page, the information the expert was looking for in the process of manual evaluation is marked in yellow. Additionally, the terms that the automated system identified as important for this criterion are boxed in different colors depending on their level of importance (eg, red=most important, green=least important).

## Results

### HONcode Principles

Table 1 gives the results of the comparison between manual evaluation and the automated system’s conformity assessment for each of the HONcode principles. When neither manual nor automated analysis found justification of conformity to a given criterion, it was considered a true negative. If a website passed both manual and automated reviews for meeting the specific criterion, it was considered a true positive. The websites where the automated detection system determined the criterion was satisfied but the expert manual evaluation disagreed were considered false positives. The websites where the automated system failed to detect a criterion even though manual review detected it were considered false negatives.

**Table 1:** Manual versus automated (using tfc and tfx weightings) evaluation (N=27).

Criteria	Manual	Automated									
		tfc					tfx				
		True^a^		False^b^		Other^c^	True^a^		False^b^		Other^c^
		–	+	–	+		–	+	–	+	
Authority	21	4	1	10	2	10	4	4	14	2	3
Complementarity	26	1	0	21	0	5	1	2	23	0	1
Privacy	24	1	14	9	2	1	3	14	10	0	0
Reference (attribution)	16	5	0	6	6	10	8	4	7	3	5
Justifiability	6	14	1	1	7	4	8	3	3	13	0
Contact details	26	1	6	16	0	4	1	15	8	0	3
Financial disclosure	17	8	1	9	2	7	9	0	16	1	1
Advertising policy	16	9	0	13	2	3	10	1	13	1	2
Date (attribution)	21	6	0	16	0	5	6	0	21	0	0

^a^ True negative: both manual and automated did not find criterion was satisfied; true positive: both manual and automated did find criterion was satisfied.

^b^ False negative: automated system did not find criterion was satisfied but manual review did; false positive: automated system did find criterion was satisfied but manual review did not.

^c^ Criterion detected on a Web page different to the one designated in the manual review.

For 23 websites, the automated system failed to detect the criterion complementarity with tfx, even though manual review detected it. In this setup, the false negatives can be interpreted as silence, whereas the false positives represent the noise.

Table 2 gives the results of the evaluation using precision and recall. In order to present the results in this form, the authors made the assumption that the results that were found on different pages between automated and manual evaluations were seen as positive detections.

**Table 2:** Precision and recall of automated HONcode detection.

Criteria	tfc		tfx	
	Precision	Recall	Precision	Recall
Authority	0.85 (11/13)	0.52 (11/21)	0.78 (7/9)	0.33 (7/21)
Complementarity	1.00 (5/5)	0.19 (5/26)	1.00 (3/3)	0.12 (3/26)
Privacy	0.88 (15/17)	0.63 (15/24)	1.00 (14/14)	0.58 (14/24)
Reference (attribution)	0.63 (10/16)	0.63 (10/16)	0.75 (9/12)	0.56 (9/16)
Justifiability	0.42 (5/12)	0.83 (5/6)	0.19 (3/16)	0.50 (3/6)
Contact details	1.00 (10/10)	0.39 (10/26)	1.00 (18/18)	0.69 (18/26)
Financial disclosure	0.80 (8/10)	0.47 (8/17)	0.50 (1/2)	0.06 (1/17)
Advertising policy	0.60 (3/5)	0.19 (3/16)	0.75 (3/4)	0.19 (3/16)
Date (attribution)	1.00 (5/5)	0.24 (5/21)	0.00 (0/0)	0.00 (0/21)

As described in Boyer and Dolamic [19], this study took the entire specific Web page as the unit of evaluation. Even though the results presented in Boyer and Dolamic indicated a high precision for automated detection of the “date” criterion, this study’s comparison had a high number of false negatives using the automated system. For this reason, the authors carried out an additional evaluation using each sentence as the evaluation unit. Table 3 gives the results of this evaluation for criteria “privacy” and “date.” Table 4 gives the results of the evaluation using precision and recall.

**Table 3:** Privacy and date criteria using sentences versus the whole document approach (N=27).

Criteria	Manual, n	Automated (tfc), n									
		Document					Sentence				
		True^a^		False^b^		Other^c^	True^a^		False^b^		Other^c^
		–	+	–	+		–	+	–	+	
Privacy	24	1	14	9	2	1	0	21	2	3	1
Date (attribution)	21	6	0	15	0	6	0	11	1	6	9

^a^ True negative: both manual and automatic did not find criterion was satisfied; true positive: both manual and automated did find criterion was satisfied.

^b^ False negative: automated system did not find criterion was satisfied but manual review did; false positive: automated system did find criterion was satisfied but manual review did not.

^c^ Criterion detected on a Web page different to the one designated in the manual review.

**Table 4:** Precision and recall of document and sentence automated HONcode detection.

Criteria	Document		Sentence	
	Precision	Recall	Precision	Recall
Privacy	0.88 (15/17)	0.63 (15/24)	0.88 (22/25)	0.92 (22/24)
Date (attribution)	1.00 (6/6)	0.24 (6/21)	0.77 (20/26)	0.95 (20/21)

### Results on the HONcode Interreviewer Agreement Level

A total of 36 websites were evaluated for each HONcode criterion by 3 HONcode senior reviewers. The results of the evaluated interrater agreement using both percent agreement and Fleiss’ kappa [25] for each of the HONcode principles are presented in Table 5.

**Table 5:** Interrater agreement, percent versus Fleiss’ kappa (κ).

Criteria	Percent agreement (%)	Fleiss’ κ	Interpretation
Authority	92.59	.745	Substantial agreement
Complementarity	79.63	–.113	Poor agreement
Privacy	85.19	.614	Substantial agreement
Reference (attribution)	88.89	.756	Substantial agreement
Justifiability	74.07	.463	Moderate agreement
Contact details	95.37	.471	Moderate agreement
Financial disclosure	87.04	.716	Substantial agreement
Advertising policy	85.19	.691	Substantial agreement
Date (attribution)	79.63	.492	Moderate agreement

## Discussion

### Principal Findings

The automated system performed the most poorly when detecting the “justifiability” criterion. Manual expert review indicated that only 6 of 27 websites fulfilled this criterion. The automated system detected this criterion for only 1 website when tfc weighting was used (eg, precision 0.42 with 4 detections on a different page), and for 3 websites with tfx (eg, precision only 0.19). Additionally, the automated system returned a large number of false positives: 7 and 13 for tfc and tfx, respectively. The poor performance of the automated system in detecting the compliance to this criterion might be explained by the fact that the data set used as a benchmark for training natural language processing algorithms for the automated detection is rather small for this criterion (eg, only 872 documents were available). In certain cases, the certain criterion might be not applicable for a given website. In that case, the website conforms to HONcode but the criteria justification will be missing from the collection. This represents the main reason of the small documents set.

When the automated system detected the criterion satisfaction on a different website page than that marked by the expert, additional manual expert review verified that the system was often correct in doing so. For example, for one website [26] the manual evaluation detected the criterion complementarity on the page [27], whereas the automated system detected it on a different page. Manual reexamination of the page on which the criterion justification was detected by the automated system confirmed that it also contained justification for satisfaction of this criterion. Even though the concept of the automated system is such that it tries to perform as close to manual evaluation as possible, a main difference exists. In the case of manual evaluation, once the criterion (eg, complementarity) is detected, not all the other pages of the website are checked. Contrarily, with the automated system, all pages are crawled before the evaluation step. Thus, the coverage can be much more important. This can also explain the detection of the criteria on other pages than that designated by the expert.

There were certain criteria, such as “date,” in which the automated system performance was unexpectedly poor. For this reason, the study examined an alternative approach using the sentence instead of the document as the classification unit (Table 3). The number of automated system detections for the criterion “date” was increased when the sentence was used as the classification unit. Similar improvements occurred using sentence-level analysis for the privacy criterion. Further studies must determine if such increases obtained using variant methods are statistically significant and should be incorporated permanently into the automatic detection algorithms. Manual analyses detected previously unknown technical problems in automated privacy criterion recognition. For one website, this particular criterion was deemed satisfied on 99% of the site’s Web pages, in addition to the page marked as correct by the expert. This did not occur when documents were used as the classification units. Another technical problem occurred when the automated system was unable to detect the date on the pages where this information was displayed using only numbers (eg, 07/07/2012) without any accompanying explanatory text. The main source of this problem was the system tokenization approach, which ignores numbers. However, changing the preprocessing and keeping the numbers in the tokenization process would not be beneficial for this criterion detection. A number can represent not only a date but also other information, which could result in a number of false positives for this and for other criteria.

As seen in Table 1, the automated system performed capably for certain criteria. The level of agreement between the manual and automated approaches elevated to 70% (eg, contact details with tfc). Such a level of agreement, approaching the 72% human agreement [28], speaks in favor of the automated system as an alternative to the manual approach. However, the system performed poorly in detecting HON principle satisfaction for funding, complementarity, date, and authority.

The privacy criterion is easy to detect for the automated system and humans. In our previous study, the automated detection of the privacy criterion showed precision of more than 92% with good recall of more than 91% [19]. However, during manual evaluation for this criterion, the expert is not only looking for the privacy statement but also verifies its implementation (eg, cookies). The automated system has to rely only on the privacy statement.

For the privacy criterion, the automated system scored 15 correct (of 24 websites that respected this criteria) for the tfc weighting scheme. Fourteen of these were true positives. It also detected criterion satisfaction on a different page than that designated by the expert for 1 website. For 2 websites, the automated system mistakenly detected privacy as satisfied. For 9 websites, the automated system failed to detect privacy satisfaction when the manual expert did so. This behavior is expected because our automated system is tuned to create less possible noise (false positives). The results described here reinforce the previous deduction of privacy criterion being the “easy” one to detect by the automated system.

Changing the weighting scheme to tfx for the privacy criterion resulted in a seeming performance enhancement. The correct results were returned for 17 websites, with no incorrect detections. This might represent random variation in study results or might suggest that the tfx method better detects the privacy criterion satisfaction.

### Manual Evaluation Interreviewer Agreement Level

In Table 5, the values of Fleiss’ kappa are rather small when compared to percent agreement. Although the values of .745 for authority and .756 for reference can be interpreted as substantial agreement, they still remain small when compared to percent agreement for these criteria. For the complementarity criterion, the kappa value of -.113 indicates disagreement in contrast to the percent agreement of 79.63% for this criterion. Two effects have been documented that might cause the misrepresentation of the interrater reliability by kappa [29]. The prevalence problem appears when one observation is coded more often than others, resulting in kappa estimation being very low, which is the case for the complementarity criterion in our study. Taking into account the particularity of the data for this criterion, kappa would not be the correct statistic to use. With a kappa value of .463, the criterion justifiability shows moderate agreement between raters (percent agreement 74%). These results show that even during the manual evaluation by experts, the criterion justifiability remains difficult to agree on. These results show that the probability of the expert giving an incorrect evaluation is quite low especially for “easy” criteria such as contact details. However, this probability is somewhat higher for more complicated criteria, such as the justifiability criterion, which further confirms the complexity of this criterion. So, this brief study identifying the level of agreement between expert reviewers shows that the automatic system behaves somewhat similarly to the manual reviewers.

### Limitation

In this evaluation, the authors compare automated HONcode conformity assessment to assessments done by a senior HONcode expert. Doing so introduces a bias. It assumes that the experts never improperly assess the presence or absence of HONcode principle satisfaction in documents. Although a HONcode expert has lower likelihood of making a false assessment than other reviewers or other automated systems, we recognize that expert assessments are not always correct, which is shown by the interrater agreement level.

### Conclusions

This study analyzed the effectiveness of an automated HONcode criteria compliance detection system. A total of 27 websites chosen with different completion statuses with respect to HONcode certification were included in the evaluation. Study results indicate a relatively high level of agreement between automatic and manual assessments for some of the HONcode criteria. Nevertheless, for other criteria, the manual approach was clearly superior. Study results suggest that “tuning” the automated detection system through future studies for each specific HONcode criterion may improve the system’s ability to detect individual criterion satisfaction. Study results also indicate that correcting a small number of technical issues in the automated system, such as the problem of not detecting the date criterion on pages displaying this information, may also improve future system performance. Incorporating third-party libraries or systems that have already proven their ability to detect and extract this kind of information [30,31] might be a solution for this issue. This approach is part of future development for this system.

The KHRESMOI project has attempted to develop a health search engine dedicated to the general public’s needs. “KHRESMOI for Everyone” (K4E) [32] is a multilingual, multimodal search and access system for biomedical information and documents. Because K4E is a specialized search engine for health information, it has specialized tools to help users to discern good quality health information from the poor quality information. K4E offers automatic detection of the 8 HONcode principles with additional trustability levels given as a percentage integrated into the search results. It also identifies the HONcode principles that are currently not being respected by the website as estimated by automatic detection so that the reader is aware of the extent to which the website can or cannot be trusted and which HONcode principle is concerned. This interface is described in detail in Pletneva et al [33]. K4E can be used in the future after further research and development based on study results conducted within the European project Kconnect [34] as a specialized quality health search engine or Web service to target trustworthy health information enabling readers to directly access this information without having to wade through multiple pages of dubious material to get there.

Another potential outcome to this study is further development of the automated detection system to assist in conducting the HONcode certification process. The present manual HONcode certification process is time consuming. Even though the level of agreement between the manual and automated systems is somewhat lower than that of 3 experts (eg, 70% vs 95% for contact details), the authors estimate that HONcode automatic detection systems might provide a first screening; thus, helping in the certification process. In summary, the future of identifying quality, trustworthy health information on the Internet will depend on development of advanced search engines with fine-tuned criterion-matching abilities that can guide users to reliable health information websites.

## Abbreviations

HON: Health on the Net
K4E: KHRESMOI for Everyone
SVM: support vector machine
